# Plasma Membrane Redox System in the Erythrocytes of Rowers: Pilot Study

**Published:** 2017-01

**Authors:** Danila Di MAJO, Valentina CONTRÒ, Antonino BIANCO, Marco GIAMMANCO, Maurizio La GUARDIA, Marcello TRAINA, Patrizia PROIA

**Affiliations:** Dept. of Psychological, Pedagogical and Educational Sciences, University of Palermo, Palermo, Italy

## Dear Editor-in-Chief

The oxidative stress results from a change in the physiological balance between oxidant and anti-oxidant species. This type of stress is a chemical change in the redox state of cells.

The increased production of reactive species is related to an excessive metabolic activation, for example, from an intense physical exercise or an excessive caloric intake (1). In physiological conditions, muscle fibers are provided with an anti-oxidant system able to keep under control the excessive production of Reactive Oxygen Species (ROS).

Endogenous and exogenous antioxidants are involved in countering the damage caused by reactive chemical species on muscle skeletal system. High-intensity training is associated with increased oxidative stress, because of the muscular effort required and the activation of all the metabolic pathways for ATP synthesis. Rowing is very demanding of high power maintained over time, mostly produced from aerobic metabolism (2).

The aim of this study was to evaluate the reducing activity in plasma and in erythrocytes in a group of rowers compared to a group of sedentary subjects, and then to evaluate the efficiency of the trans-plasma membrane electron transport (TPMET), also known as PMRS, in the erythrocytes of rowers as a compensatory mechanism of cellular redox homeostasis. Twenty-six healthy volunteers who did not practice physical activity at a competitive level (control subjects group) (26.6±2.2 yr) and twenty-two professional rowers were recruited (18.6±3.1 yr). Venous blood samples were collected from rowers and control group after overnight fast. After centrifugation at 3000 rpm for 10 min at 4 °C, plasma was separated from red blood cells. The resultant plasma was transferred to micro centrifuge tubes and used at least in part for ferric-reducing activity power (FRAP) assay. Red blood cells, after removal of buffy coat and upper 15% of the packed red blood cells, were washed two times with cold PBS (3). The ferric reducing activity in plasma and erythrocytes (FRAP) assay was performed (4). The PMRS activity was evaluated by reduction of 1 mM-ferricyanide (FIC) solution in PBS (pH 7.4) in ferrocyanide (FOC), using 1, 10-phenanthroline as an indicator and measuring absorption at 510 nm. All analyses were performed using GraphPad Prism 5 (GraphPad Software Inc., Chicago, IL, USA). The statistical significance level was set at *P*<0.05.

The main finding of this study concern the activity of plasma membrane redox system (PMRS): it has been elevated of 35% in RBCs from rowers than in RBCs from untrained group. The PMRS operated to maintain the ascorbate level in plasma, concerning the compensatory/protective mechanism. The plasma membrane redox system activity varies from 443 to 475μmoL ferrocyanide/mL PRBC/30 min vs the range of 281 to 311 μmoL ferrocyanide/mL PRBC/30 min of the control group. The antioxidant status (FRAP) measured in plasma and in erythrocytes as well as plasma membrane redox system (PMRS) activity for untrained and rower groups are shown in Table 1. Plasma antioxidant capacity levels, measured by FRAP, was 21% lower in rowers group compared to untrained one (*P*=0.02).

**Table 1:** Plasma and erythrocyte antioxidant status and membrane redox of system values of the study partecipants

**Parameter**	**Untrained subjects (n= 26)**	**Rowers (n=22)**	***P*-value**
* Plasma *			
FRAP (μmol/L)	581 ± 161	460 ± 81	0.02*
* Erythrocytes *			
FRAP (μmol/L)	1691 ± 128	1818 ± 401	0.17
PMRS (μmol ferrocyanide/mL PBRC/30min)	2,96 ± 0,15	4,59 ± 0,16	<0.0001*

Data are expressed as means ± SD. FRAP, ferric reducing ability of plasma and of erythrocytes; PMRS, Plasma Membrane Redox System;

* Significant difference by unpaired Student’s t-Test

Definitely, athletes who play maximal aerobic/anaerobic sports have a higher NADH/NADPH level compare with sedentary subjects. In athletes engaged in exhaustive exercise like rowing, there was a significant increase in the oxidative phosphorylation, necessary to support a higher demand of ATP (5).

We concluded that, despite extreme exercise like rowing cause an increase of oxidant species production, well-trained athletes that following a healthy lifestyle do not show an oxidative stress condition because intensive but constant training determines an adaptation of the antioxidant system that becomes more efficient, minimizing the redox imbalances.
